# Acox2 is a regulator of lysine crotonylation that mediates hepatic metabolic homeostasis in mice

**DOI:** 10.1038/s41419-022-04725-9

**Published:** 2022-03-29

**Authors:** Yuan Zhang, Yuling Chen, Zhao Zhang, Xiang Tao, Sha Xu, Xinyan Zhang, Tinatin Zurashvili, Zhouping Lu, José Ramon Bayascas, Liping Jin, Jianyuan Zhao, Xiangyu Zhou

**Affiliations:** 1 Obstetrics and Gynecology Hospital of Fudan University, School of Life Sciences, Fudan University, 200011 Shanghai, China; 2 Shanghai Key Laboratory of Maternal Fetal Medicine, Department of Assisted Reproduction, Shanghai First Maternity and Infant Hospital, Tongji University School of Medicine, 201204 Shanghai, China; 3 State Key Lab of Genetic Engineering, School of Life Sciences, Fudan University, 200438 Shanghai, China; 4 School of Basic Medicine, Fudan University Shanghai Medical College, 200032 Shanghai, China; 5 Department of Biochemistry, David Tvildiani Medical University, Tbilisi, 0159 Georgia; 6 Departament de Bioquímica i Biologia Molecular, Institut de Neurociències, Universitat Autònoma de Barcelona, Barcelona, 08193 Spain; 7 Institute for Developmental and Regenerative Cardiovascular Medicine, MOE-Shanghai Key Laboratory of Children’s Environmental Health, Xinhua Hospital, Shanghai Jiao Tong University School of Medicine, 200092 Shanghai, China

**Keywords:** Metabolic disorders, Post-translational modifications

## Abstract

Acyl-CoA oxidase 2 (Acox2) is an enzyme involved in peroxisomal bile acid synthesis and branched-chain fatty acid degradation. *Acox2* knockout (−/−) mice spontaneously developed liver cancer with marked lymphocytic infiltrate. Tandem-affinity purification coupled with mass spectrometry analysis revealed that Acox2 interacted with methylcrotonoyl-CoA carboxylase followed by co-immunoprecipitation confirmation. Here we reported that non-histone lysine crotonylation (Kcr) levels were downregulated in *Acox2*^*−/−*^ mice livers. Interestingly, Kcr signals were concentrated in the nucleus of tumor cells but mostly located in the cytoplasm of adjacent normal liver cells of *Acox2*^−/−^ mice. Quantitative analysis of the global crotonylome further revealed that 54% (27/50) of downregulated non-histone Kcr sites were located in mitochondrial (11/50) and peroxisomal (17/50) enzymes including Ehhadh, Scp2, Hsd17b4, Crot, Etfa, Cpt1a, Eci1/2, Hadha, Etfdh, and Idh2. Subsequent site-directed mutagenesis and transcriptome analysis revealed that Ehhadh K^572^cr might have site-specific regulatory roles by downregulating TOP3B expression that lead to increased DNA damage in vitro. Our findings suggested Acox2 is a regulator of Kcr that might play critical role on hepatic metabolic homeostasis.

## Introduction

Beta-oxidation chain shortening of very-long-chain fatty acids, 2-methyl-branched fatty acids, and the CoA esters of C27 bile acid intermediates di- and trihydroxycoprostanoic acid occur in the peroxisomes [1, 2]. There are four enzymatic steps in each cycle of peroxisomal β-oxidation, including oxidation, hydration, dehydrogenation, and thiolytic cleavage [3]. Acyl-CoA oxidases (ACOXs) catalyze the first step. Three ACOX1/2/3 with different substrate specificities are found in humans [4]. ACOX1 is responsible for the oxidation of very long straight-chain fatty acids [5], ACOX2 is the only acyl-CoA oxidase involved in bile acid biosynthesis, and both ACOX2 and ACOX3 are involved in the degradation of branched-chain fatty acids [4, 6]. ACOX2 is predominantly expressed in the liver and kidney, whereas ACOX3 has relatively lower expression levels in the liver. In the peroxisomes, D-bifunctional enzyme (HSD17B4) or L-bifunctional enzyme (EHHADH) catalyze the second and third steps of β-oxidation, respectively [7]. The last step in beta-oxidation can be performed by multiple thiolases, including acetyl-CoA acyltransferase 1 (ACAA1), and the sterol carrier protein x (SCPx or SCP2) [8]. Then shortened fatty acids are transported to the cytosol for mitochondrial uptake through the action of carnitine octanoyltransferase (CROT) [9].

Lysine crotonylation (Kcr) has emerged as a key posttranslational modification that plays regulatory roles in many biological processes [10–12]. Interestingly, a recent study reported that both ACOX1 and ACOX3 were key crotonyl-CoA-producing enzymes, and deletion of ACOX3 in hESCs led to reduced Kcr, which impaired mesoderm or endoderm differentiation [13]. Short-chain enoyl–CoA hydratase (ECHS1), an enzyme involved in mitochondrial fatty acid beta-oxidation, controls intracellular crotonyl-CoA and maintains the maturity and homeostasis of cardiomyocytes through histone crotonylation [14]. These findings suggested that mitochondrial and peroxisomal fatty acid oxidation was important for crotonyl-CoA production. On the other hand, the classic histone acetyltransferases p300, pCAF, and MOF also have histone crotonyltransferase activity [15, 16], whereas the classic histone deacetylases HDAC1/2/3 and SIRT1/2/3 can remove Kcr under different conditions [15, 17]. Many non-histone proteins are also modified by crotonylation [18]. However, the functional elucidation of non-histone Kcr is in its infancy.

We previously reported that Acox2 deficiency is associated with a metabolic switch in primary malignant cardiac tumor progression [19, 20]. ACOX2 homozygous stop-gain (p.Y69*) and missense (p.R225W) mutations have been identified in patients with transaminase elevation and liver fibrosis [21, 22]. We generated an *Acox2* knockout mice model that exhibited the typical clinical symptoms of fatty liver disease at the age of 4 months [23]. Notably, nicotinamide *N*-methyltransferase (NNMT), a critical regulator of metabolism and epigenetics [24, 25], and its product 1-methylnicotinamide were uniformly downregulated in both serum and liver tissues of *Acox2*^−/−^ mice [23]. Since Acox2 and Acox3 have similar function in peroxisomal β-oxidation; therefore, hepatic Kcr was presumed to be downregulated in *Acox2*^−/−^ mice. In this study, we perform integrative analysis and find that Acox2 is a master regulator for protein post-translational modifications that involved in the metabolic homeostasis.

## Results

### Acox2 loss induces liver cancer in mice

**Fig. 1:**
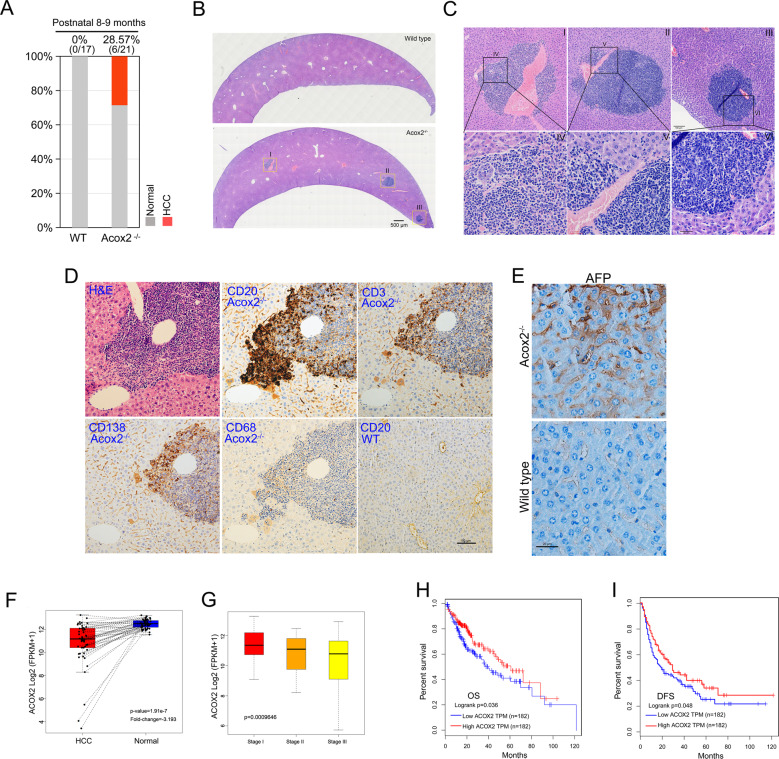
Acox2 loss induces HCC in mice. **A** Percentage distribution of liver cancer was compared between *Acox2*^−/−^ (*n* = 21) and WT (*n* = 17) mice at the age of 8–9 months. **B** The representative image of HE stained cross-sections of liver tissue in *Acox2*^−/−^ and WT mice. Scale bar, 500 μm. **C** Three tumor lesions (I–III) from a single cross-section of *Acox2*^−/−^ mouse liver are boxed to show the enlarged region (IV–VI) as indicated. Scale bars, 100 μm in upper panel and 30 μm in bottom panel. **D** Staining was performed using HE and immunostaining against CD3, CD20, CD68, and CD138 on liver paraffin sections of Acox2^−/−^ or WT mice. Representative images were displayed as indicated. Scale bar, 100 μm. **E** Immunohistochemical staining of formalin-fixed paraffin-embedded section of mouse liver tissues using AFP antibody. Representative images were displayed as indicated. Scale bar, 20 μm. **F**, **G** ACOX2 expression level in humans is significantly downregulated in HCC than in normal liver specimens (fold-change = −3.193; *p*-value = 1.91 × 10^−7^) (**F**) and decreases gradually in malignant stage (III) when compared with the early stage (I) (*p*-value = 0.00096) (**G**) by analyzing data from TCGA portal. **H**, **I** Low ACOX2 expression in human HCC is correlated with a decrease in OS (overall survival, *p*-value = 0.036) (**H**) and DFS (disease free survival, *p*-value = 0.048) (**I**) when comparing to high ACOX2 expression as observed on analyzing data from TCGA portal.

We generated an Acox2 knockout C57BL/6n mouse model using the CRISPR/Cas9 gene editing system. A deletion of 15,146 bp with a span from exon 3 to exon 13 was identified (Supplementary Fig. S1A) in Acox2 knockout (-/-) mice. Importantly, Hematoxylin and eosin (H&E) staining revealed that 28.57% (6/21) Acox2^−/−^ mice spontaneously developed liver cancer without high-fat diet induction or small molecule treatment at the postnatal age of 8–9 months (Fig. 1A–C and S1B), whereas wild type (WT) mice did not develop such tumors. Inflammatory infiltration is very popular in the liver tissues of Acox2^−/−^ mice. We performed immunohistochemical stains for immune cell markers (CD3, CD20, CD138, and CD68) to determine the immune cells types and immunocyte infiltration characteristics. The predominant cell-type is B-lymphocytes CD20, followed by T-lymphocytes CD3 and plasma cells CD138, but is almost negative for macrophages CD68 (Fig. 1D). There was a co-appearance of CD3 and CD20. Compared with WT mice, we also observed positive staining for Alpha-fetoprotein (AFP) in Acox2^−/−^ mice (Fig. 1E). Heterogeneity was identified in tumor lesion of Acox2^−/−^ mice liver (Fig. S1B). In addition, the ACOX2 expression level in humans is significantly downregulated in HCC than in normal liver specimens (fold-change = −3.19; *p*-value = 1.91 × 10^−7^) (Fig. 1F) and decreases gradually in malignant stages when compared with the early stages (*p*-value = 0.00096) (Fig. 1G). These findings are further supported by low ACOX2 expression in human HCC is correlated with a decrease in OS (overall survival, *p*-value = 0.036) (Fig. 1H) and DFS (disease free survival, *p*-value = 0.048) (Fig. 1I) when comparing to high ACOX2 expression as observed on analyzing data from TCGA portal.

### Acox2 Interacts with methylcrotonoyl-CoA carboxylase

**Fig. 2:**
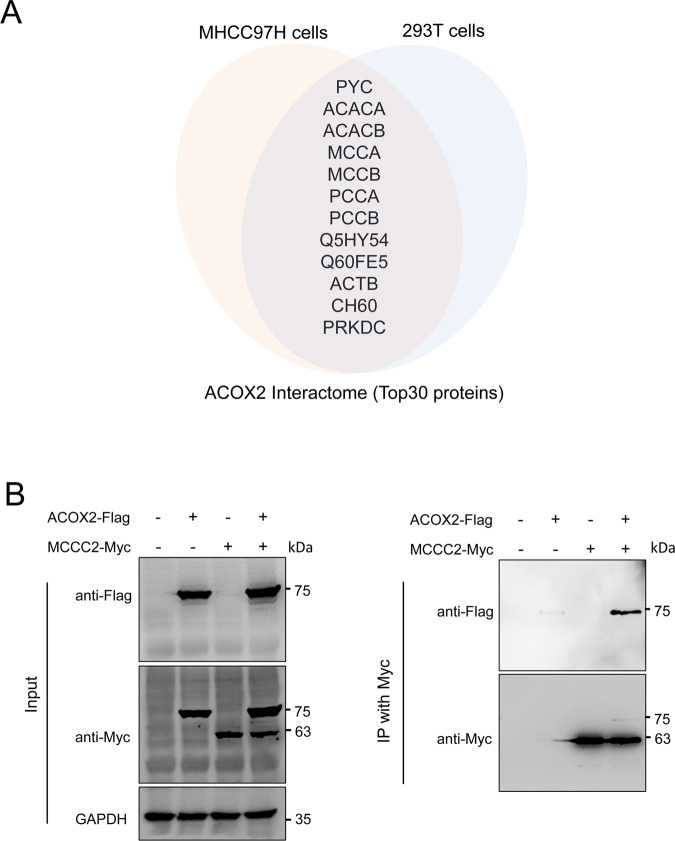
Acox2 interacts with methylcrotonoyl-CoA carboxylase. **A** Overlapped Acox2-interacting proteins in MHCC97H and HEK293T cells by TAP-MS analysis were displayed. **B** Anti-Myc immunoprecipitation assays were performed in HEK293T cells overexpressing Flag-tagged ACOX2 and Myc-tagged MCCC2 constructs followed by immunoblotting with the indicated antibodies. Specificity of antibodies was evaluated as indicated (left panel). MCCC2 methylcrotonoyl-CoA carboxylase subunit 2, MS mass spectrometry, TAP tandem affinity purification.

We next used tandem-affinity purification coupled with mass spectrometry (TAP-MS) analysis to determine the interactome of ACOX2 and its functions in MHCC97H and HEK-293T cell lines, respectively (Supplementary Tables S1 and S2). Since ACOX2 works as a homodimer that is essential for biological function, ACOX2 was also identified as an interacting partner in both cell lines, which further validate the data reliability of TAP-MS analysis. Approximately 40% (12/30) of the top 30 interacting proteins for Acox2 overlapped in MHCC97H and HEK-293T cells (Fig. 2A). In particular, four classic biotin-dependent carboxylases [26], including acetyl-CoA carboxylase 1 (ACACA) and 2 (ACACB), and methylcrotonoyl-CoA carboxylase subunit alpha (MCCA) and beta chain (MCCB), propionyl-CoA carboxylase subunit alpha (PCCA) and subunit beta (PCCB), and pyruvate carboxylase (PC), have protein–protein interactions with ACOX2. The interaction between ACOX2 and MCCC2 (MCCB) was further confirmed by co-immunoprecipitation in HEK-293T cells (Fig. 2B). MCCC1 and MCCC2 could form a heterodimer and catalyze the carboxylation of 3-methylcrotonyl-CoA to 3-methylglutaconyl-CoA [27], and intracellular crotonyl-CoA could directly regulate Kcr [12]. In addition, ACOX2 has protein interaction with DNA topoisomerases including TOP2A and TOP2B in MHCC97H cells (Table S1).

### Kcr changes are associated with liver cancer progression in Acox2^−/−^ mice

**Fig. 3:**
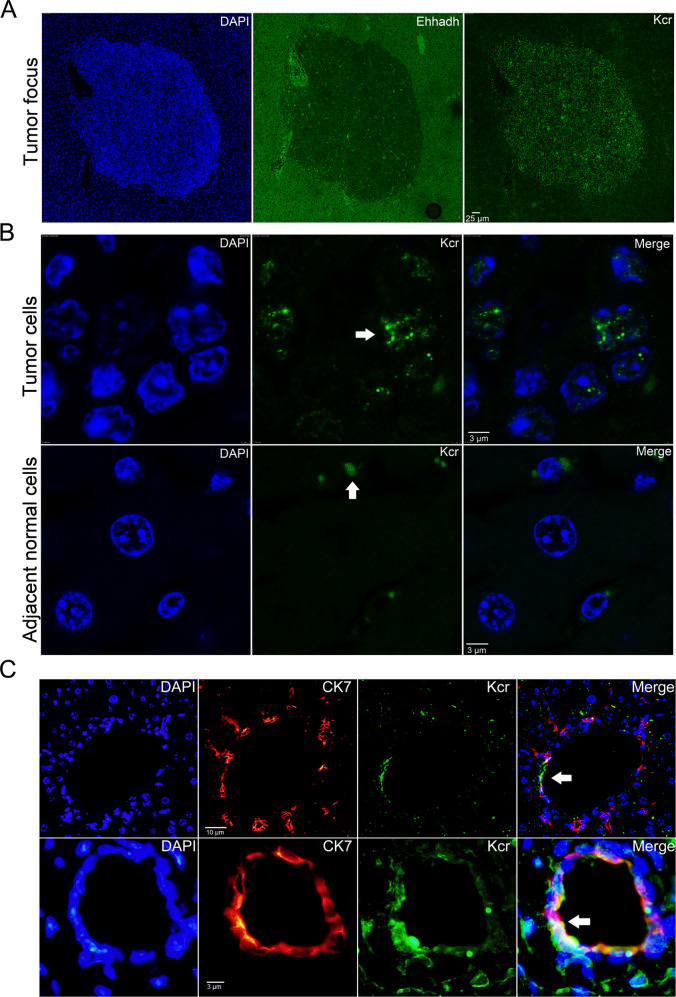
Kcr levels are associated with HCC progression in Acox2^−/−^ mice. **A** Fluorescent immunohistochemistry using DAPI, anti-Ehhadh and pan anti-Kcr were performed on three slides of a single tumor focus. Scale bar, 25 μm. **B** Immunohistochemical staining showed Kcr signals were concentrated in the nucleus of tumor cells (upper panel) but mainly located in the cytoplasm of adjacent normal liver cells (bottom panel) in *Acox2*^*−/−*^ mice, Scale bar, 3 μm. **C** Kcr levels were increased in the regions of bile duct proliferation in *Acox2*^*−/*−^ mice by double immunostaining with Kcr and a ductal marker CK7. Co-localization of Kcr signal with CK7 was observed and indicated by white arrow. Representative images were displayed. Scale bar, 10 μm (upper) and 3 μm (bottom).

To investigate the potential correlation between Kcr levels and HCC progression, we examined Kcr changes in tumor and adjacent normal cells of Acox2^−/−^ mice. A previously well-characterized rabbit pan-anti-Kcr antibody (PTM-501) was utilized to perform immunofluorescence staining [13, 28]. To further clarify the specificity of this Kcr antibody, we firstly evaluated the distribution and levels of Kcr in two cultured cell lines including iPSC (induced pluripotent stem cells) and HepG2. We observed strong positive signals in both nucleus and cytoplasm of iPSCs (Fig. S2A), which is consistent with the findings from the previous study [13]. Meanwhile, we found that the Kcr signals are mostly concentrated in the nucleus of HepG2 cells (Fig. S2A). Since KATs and HDACs can modulate histone Kcr levels [29, 30], HepG2 cells were then treated with p300 inhibitors and HDACIs, respectively. Compared to cells without treatment (Ctrl), we identified decreased Kcr levels in cells treated with C646 (2 μm) whereas Kcr signals were upregulated in cells treated with FK228 (0.25 μM) by immunoblotting (Fig. S2B) and immunofluorescence assay (Fig. S2C). These findings suggested that pan-anti-Kcr antibody provided adequate sensitivity for detecting Kcr changes in Acox2^−/−^ mice. In this study, we found that Kcr levels were more concentrated in the nucleus of tumor cells (Figs. 3A, B and S3) whereas adjacent normal liver cells that harbor most of Kcr signals in the cytoplasm through confocal immunofluorescence microscopy. By contrast, levels of Ehhadh were markedly downregulated in the tumor lesions of Acox2^−/−^ mice (Fig. 3A), which further validates the specificity of the fluorescence signal for the anti-Kcr antibody. In addition, Kcr fluorescence signal intensity in cytoplasm of normal liver cells in Acox2^−/−^ mice was significantly decreased when compared with WT mice (Fig. S4A). Since Acox2^−/−^ mice displayed bile duct proliferation (Fig. S4B), we also evaluate the possible Kcr changes in areas of ductular reaction. Staining for Sox9 and CK7 was used to outline bile ducts and ductular reaction as previous studies [31–33]. We found that Kcr levels were increased in areas of ductular reaction when compared with adjacent liver tissues (Figs. 3C and S4C). Co-localizations of Kcr signal and ductal markers (CK7 and Sox9) were observed by double immunostaining (Figs. 3C and S4D). These findings suggest that Kcr might play important roles in liver cancer progression.

### Downregulation of non-histone Kcr in Acox2 mice

**Fig. 4:**
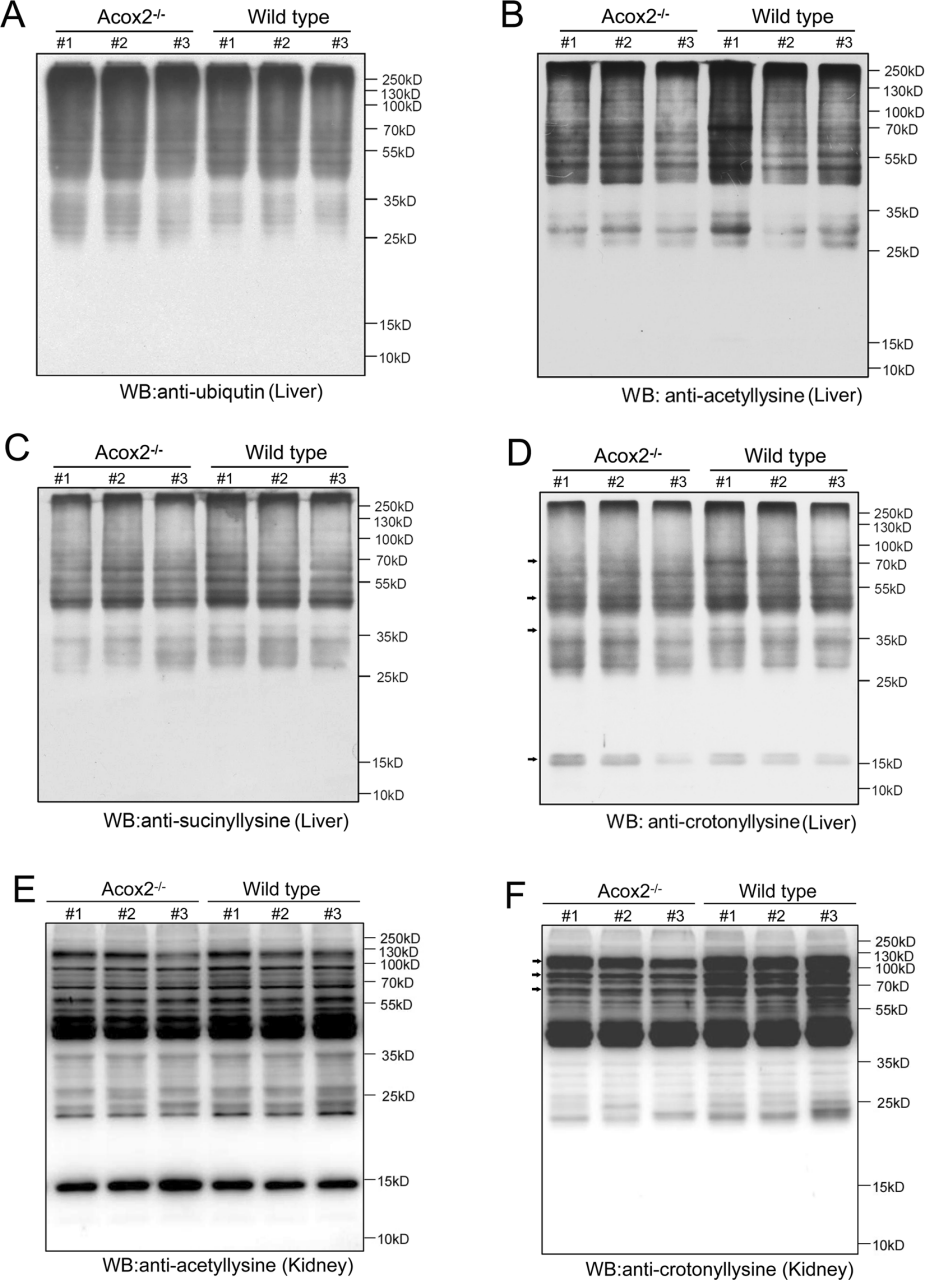
Downregulation of non-histone Kcr in Acox2^−/−^ mice. **A**–**D** Overview of anti-ubiquitin (**A**), -succinyl lysine (**B**), -acetyl lysine (**C**), and –crotonyl lysine (**D**) in liver tissues of *Acox2*^−/−^ (*n* = 3) and WT (*n* = 3) mice using corresponding pan-antibodies by western blots. **E**, **F** Overview of anti-acetyl lysine (**E**), and –crotonyl lysine (**F**) in kidney tissues of *Acox2*^−/−^ (*n* = 3) and WT (*n* = 3) mice using corresponding pan-antibodies by western blots.

Since Acox2 and Acox3 have overlapped function on peroxisomal β-oxidation, we postulated that Kcr levels in Acox2^−/−^ mice might be changed. To address this concern, an overview of four different classic protein post-translational modifications, including ubiquitination (Fig. 4A), acetylation (Fig. 4B), succinylation (Fig. 4C), and crotonylation (Fig. 4D), was obtained through western blot analysis using the corresponding pan antibody. We did not observe significant differences in acetylation, ubiquitination, and succinylation between liver tissues of Acox2^−/−^ mice and their WT littermates. However, non-histone lysine crotonylation (Kcr) was partially downregulated in the liver tissue of Acox2^−/−^ mice when compared with wild-type mice (Fig. 4D). By contrast, a mild increase in histone Kcr was observed in Acox2^−/−^ mice. In addition, decreased non-histone Kcr was also detected in the kidney tissues of Acox2^−/−^ mice (Fig. 4F) whereas acetylation (Fig. 4E) was not significantly changed.

### Histone (H2b) K86cr was upregulated in Acox2^−/−^ mice

**Fig. 5:**
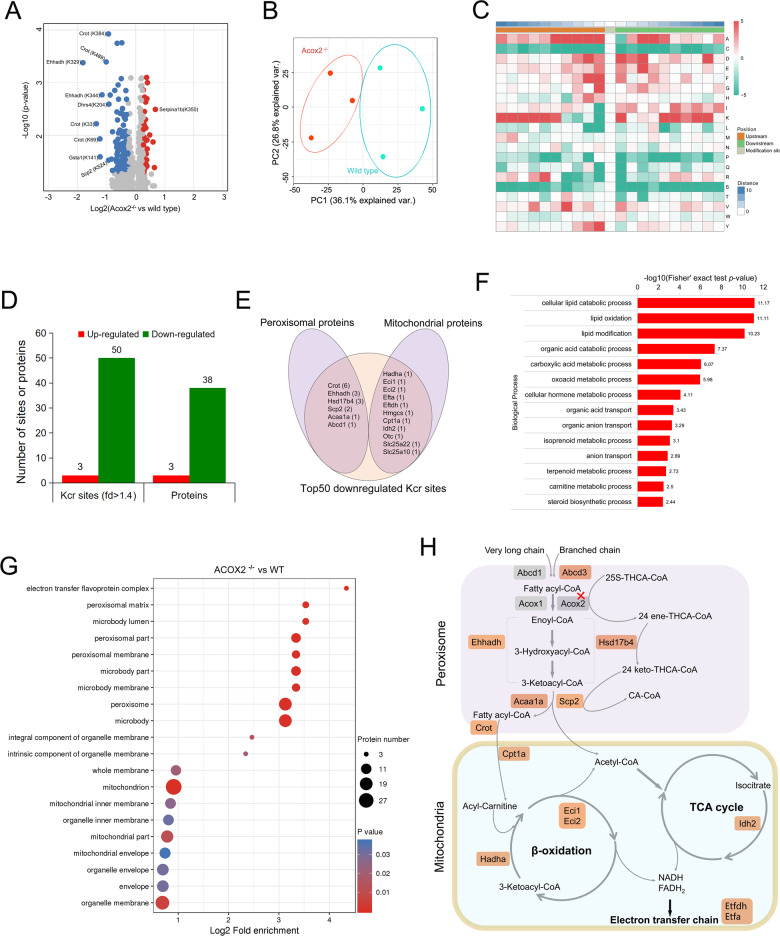
Global crotonylome analysis of liver tissues in Acox2^−/−^ and WT mice. **A** Volcano plots showing the significantly changed Kcr sites (FDR < 1%, *p* < 0.05). Up- and downregulated Kcr sites are highlighted in red and blue, respectively. **B** Score plots of principal component analysis (PCA) of *Acox2*^−/−^ and WT crotonylome. **C** Motif analysis of all identified crotonylated sites. **D** Compared with the wild type (WT) group, 50 downregulated Kcr sites on 38 proteins and 3 upregulated Kcr sites on 3 proteins were identified in *Acox2*^−/−^ mice (fold-change >1.4; FDR < 1%; *p* < 0.05). **E** Venn diagram showing 32% (16/50) and 22% (11/50) of downregulated Kcr sites were located in the proteins involved in peroxisomal and mitochondrial metabolism, respectively. **F**, **G** Analysis of Biological Process (**F**) and Cellular Component (**G**) of downregulated Kcr proteins by Gene Ontology (GO) enrichment (Fisher’s exact test; *p* < 0.05). Top selected pathways are shown in each category as indicated. **H** Schematic representation of downregulated hepatic Kcr on enzymes involved in fatty acid oxidation and TCA cycle in *Acox2*^−/−^ mice. Proteins carrying downregulated Kcr sites are marked in brown.

To precisely determine hepatic Kcr changes caused by *Acox2* knockout, global crotonylome analysis was performed using the liver tissues of *Acox2*^−/−^ (*n* = 3) and WT littermates (*n* = 3) that were collected at four months of age (Fig. 5A). Principal component analysis (PCA) separated the six liver tissue samples into two distinct categories (Fig. 5B). All 2324 quantifiable crotonylation sites in 848 proteins were normalized according to the corresponding quantitative proteomics analysis for each sample. We found that Kcr is prevalent in mouse liver and almost every enzyme that catalyzes intermediate metabolism, including fatty acid oxidation, the TCA cycle, gluconeogenesis, the urea cycle, glycogen metabolism, and glycolysis, was crotonylated. We next analyzed the flanking sequences of these sites and found that aspartic acid (D) and phenylalanine (F) were overrepresented, whereas glutamine (Q), arginine (R), and cysteine (C) were less than expected at the −1 and + 1 positions surrounding the lysine crotonylation sites (Fig. 5C).

Crotonylome analysis identified a total of 23 Kcr sites on histone proteins, including histone (H)1.4 (Hist1h1e), H2A (H2afj, H2afx, Hist2h2ac, and Hist2h2bb), H2B (Hist1h2bc and Hist1h2bp), H3.3 (H3f3a), and H4 (Hist1h4a). Among them, 60.87% (14/23) of Kcr sites were quantifiable (Table 1). We found that H2bc4 (K86cr) was the only site that could meet our filter strategy (fold-change > 1.4, *p* < 0.05) that is significantly upregulated in Acox2^−/−^ mice. However, classic H2b clustered histone 4 (H2bc4) (K12cr) was not significantly changed in Acox2^−/−^ mice in our study. These findings suggest that histone and non-histone Kcr might be differentially regulated in Acox2^−/−^ mice livers.

**Table 1:** 14 Quantifiable Kcr sites on histone proteins were identified in Acox2^−/−^ mice by global crotonylome.

Gene name	Histone protein description	Amino acid	Kcr sites	Kcr Ratio (Acox2^−/−^ _vs_ WT)	*p*-value
H2afx	Histone H2AX	K	134	0.968	0.43172
H3f3a	Histone H3.3	K	80	0.911	0.38218
Hist1h1e	Histone H1.4	K	106	0.939	0.2908
Hist1h2bc	Histone H2B type 1-C/E/G	K	35	0.96	0.39846
Hist1h2bc	Histone H2B type 1-C/E/G	K	21	0.969	0.84268
**Hist1h2bc**	**Histone H2B type 1-C/E/G**	**K**	**86**	**1.511**	**0.005361**
Hist1h2bc	Histone H2B type 1-C/E/G	K	12	0.985	0.92898
Hist1h2bc	Histone H2B type 1-C/E/G	K	121	1.025	0.73184
Hist1h2bc	Histone H2B type 1-C/E/G	K	47	1.108	0.132937
Hist1h2bp	Histone H2B type 1-P	K	12	1.139	0.0129956
Hist1h4a	Histone H4	K	78	0.888	0.112897
Hist2h2ac	Histone H2A type 2-C	K	37	0.954	0.73574
Hist2h2ac	Histone H2A type 2-C	K	96	0.907	0.062754
Hist2h2bb	Histone H2B type 2-B	K	21	1.015	0.82528

Bold values represent the significantly upregulated Kcr site (fold-change >1.4, *p*-value < 0.05).

### Downregulated non-histone Kcr sites on metabolic enzymes

For non-histone proteins, a total of 50 downregulated Kcr sites on 38 proteins and 3 upregulated Kcr sites on 3 proteins were identified in *Acox2*^−/−^ mice (fold-change > 1.4; *p* < 0.05) when compared with the WT group (Fig. 5D and Table S3), which further confirmed the downregulation of non-histone Kcr in *Acox2*^−/−^ mice. Approximately 32.0% (16/50) of downregulated Kcr sites were located in the proteins of peroxisomal β-oxidation pathway including Crot (K[lys]^33^, K^69^, K^143^, K^226^, K^384^, and K^489^), Hsd17b4 (K^57^, K^68^, and K^81^), Ehhadh (K^329^, K^344^, K^572^), Scp2 (K^211^ and K^524^), Acaa1a (K^292^), and Abcd3 (K^529^) (Fig. 5E). Moreover, 22.0 % (11/50) of downregulated Kcr sites were located in mitochondrial proteins, including Hadha (K^190^), Eci1 (K^229^), Eci2 (K^90^), Cpt1a (K^180^), Otc (K^80^), Idh2 (K^67^), Slc25a22 (K^188^), Slc25a10 (K^253^), Etfa (K^139^), Etfdh (K^152^), and Hmgcs2 (K^327^) (Fig. 5E). Analysis of Biological Process (BP) and Cellular Component (CC) of downregulated Kcr proteins by Gene Ontology (GO) revealed that “cellular lipid catabolic process,” “lipid oxidation,” and “lipid modification” were the top 3 enriched terms for BP analysis (Fig. 5F), and “peroxisome,” “peroxisomal part,” and “mitochondrion” were the top 3 enriched terms for CC analysis (Fig. 5G). These findings reveal the key regulatory roles of non-histone Kcr on peroxisomal and mitochondrial metabolism in mouse liver (Fig. 5H).

### Validation of Kcr changes on Ehhadh and Crot

**Fig. 6:**
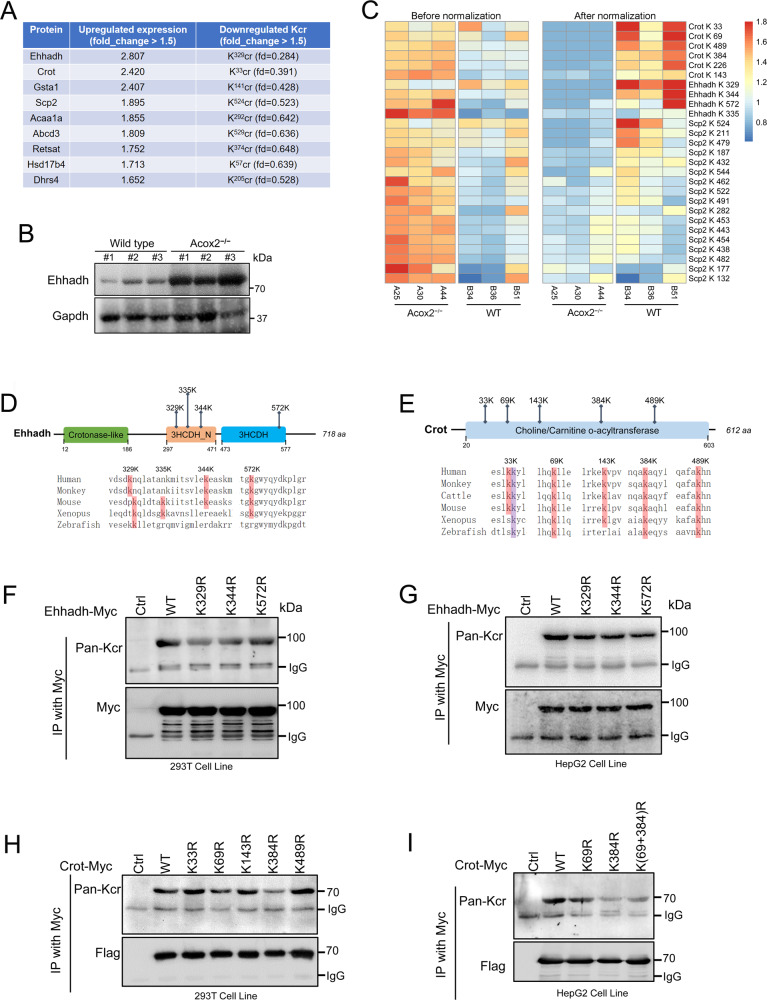
Downregulated non-histone Kcr sites on metabolic enzymes. **A** Key peroxisomal enzymes including Ehhadh, Scp2, Crot, and Hsd17b4 carrying downregulated Kcr sites were significantly upregulated (fd > 1.5) in *Acox2*^−/−^ mice. **B** Upregulation of Ehhadh expression in *Acox2*^*−/−*^ mice was verified through western blotting. **C** Heatmap showing profiles of all quantifiable Kcr sites in Crot, Ehhadh, and Scp2 before or after normalization according to quantitative proteomics analysis. **D**, **E** Sequence alignment of identified Kcr sites in Ehhadh (**D**) or Crot (**E**) in different species including xenopus and zebrafish. **F**, **G** Replacing lysine with arginine at Kcr sites lead to decreased Ehhadh Kcr levels in 293T (**F**) and HepG2 (**G**) cells as indicated. Cells were transfected with Myc-tagged WT and point mutants of Ehhadh. Cell lysates were immunoprecipitated with an anti-Myc antibody followed by immunoblotting with a pan-Kcr antibody. **H**, **I** Replacing lysine with arginine at Kcr sites leads to decreased Crot Kcr levels in 293T cells (**H**) and HepG2 cells (**I**). Representative image from three independent experiments was displayed.

In our study, the most significantly changed Kcr sites were mainly located at key enzymes in peroxisomal beta-oxidation including Ehhadh, Crot, Scp2, Hsd17b4, Acaa1a and Abcd3. Interestingly, protein levels of these enzymes carrying a number of downregulated Kcr sites were significantly upregulated in *Acox2*^−/−^ mice (Fig. 6A, B). All six Kcr sites in Crot, including K^33^, K^69^, K^489^, K^384^, K^226^, and K^132^, were downregulated by 0.391-, 0.426-, 0.494-, 0.522-, 0.544-, and 0.664-fold, respectively. Ehhadh K^329^, K^344^, and K^572^ decreased by 0.284-, 0.448-, and 0.621-fold, respectively, whereas K^335^ did not significantly change (fold-change = 0.906) (Fig. 6C). A total of 17 Kcr sites were identified in Scp2. Sequencing alignment indicated that Ehhadh K^329^ and K^572^ (Fig. 6D) and Crot K^69^, K^384^ and K^489^ (Fig. 6E) were highly conserved across species, including zebrafish or xenopus. We then generated Crot or Ehhadh mutants in which lysine Kcr sites were simultaneously mutated to arginine by site-specific mutagenesis to mimic a decrotonylated status. Crotonylation of Ehhadh (Fig. 6F, G) and Crot (Fig. 6H, I) was further confirmed by using immunoprecipitation assays in HEK-293T and HepG2 cell lines overexpressing Myc-tagged Ehhahdh or Crot recombinant proteins, respectively. Mutating lysine to arginine in Kcr sites of Crot (K^69^R and K^384^R) or Ehhadh (K^329^R, K^344^R and K^572^R) reduces Kcr levels to various degrees in vitro (Fig. 6F–I), which further validate the data reliability of crotonylome analysis.

### Site-specific regulatory role of Ehhadh Kcr on the transcriptome in vitro

**Fig. 7:**
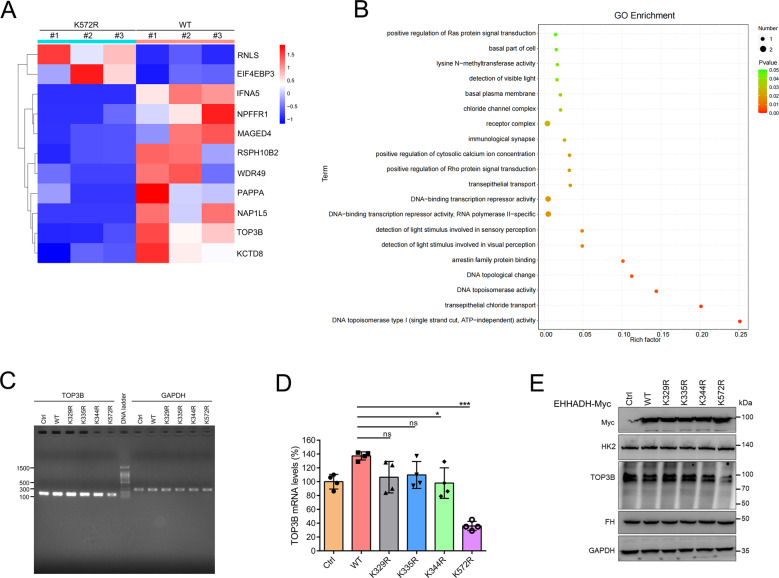
Site-specific regulatory role of Ehhadh K572cr on the transcriptome in vitro. **A** Hierarchical clustering and Heatmap showing the profiles of differentially expressed genes (DEGs) in 293T cells expressing Ehhadh K572R when compared with Ehhadh WT. **B** Gene Ontology enrichment analysis revealed that “DNA topoisomerase type I (single strand cut. ATP-independent) activity” was the most significantly enriched term in the K572R vs WT group. **C**, **D** mRNA levels of TOP3B were further confirmed by cDNA PCR (**C**) and quantitative real-time PCR (**D**). *p < 0.05; ***p < 0.001 (one-way ANOVA test, *n* = 4). **E** Western blot showed protein levels of TOP3B was downregulated whereas expression of FH and HK2 was not affected in cells overexpression K572R when compared with WT and other mutants. Representative image from three independent experiments was displayed.

Ehhadh K329cr (fd = 0.284) and K335cr (fd = 0.906) were differentially regulated in *Acox2*^−/−^ mice, which implicates the possible site-specific regulatory role of Ehhadh Kcr on biological processes. To mimic the upregulation of Ehhadh and investigate the site-specific effects of Kcr changes, HEK-293T cells were transfected with control (Ctrl) and Ehhadh WT or mutants (K to R) recombinant Myc-tagged plasmids, as indicated. Overexpression of Ehhadh was validated through western blotting. We next performed RNA-seq based transcriptome analysis with three biological replicates for each sample. Subsequent PCA analysis based on 3 biological replicates effectively separated K572R from Ehhadh WT or the other mutants (Fig. S5A). Hierarchical clustering results and heatmap representation of DEGs profiles are shown (Figs. 7A and S5B). Of note, Top3b (DNA topoisomerase III beta) was significantly downregulated in cells expressing Ehhadh K572R when compared with WT. GO enrichment analysis revealed that “DNA topoisomerase type I (single strand cut, ATP-independent) activity” was the most significantly enriched term on site-specific DEGs from K572R probably due to the downregulation of DNA topoisomerase III beta (TOP3B) (Fig. 7B). Subsequent cDNA amplification (Fig. 7C), real-time (RT) quantitative PCR (Fig. 7D), and western blot (Fig. 7E) uniformly confirmed that TOP3B expression was downregulated in cells transfected with Ehhadh K572R when compared with Ehhadh WT and other mutants. These findings suggest that non-histone Kcr has site-specific regulatory roles in biological processes.

**Fig. 8:**
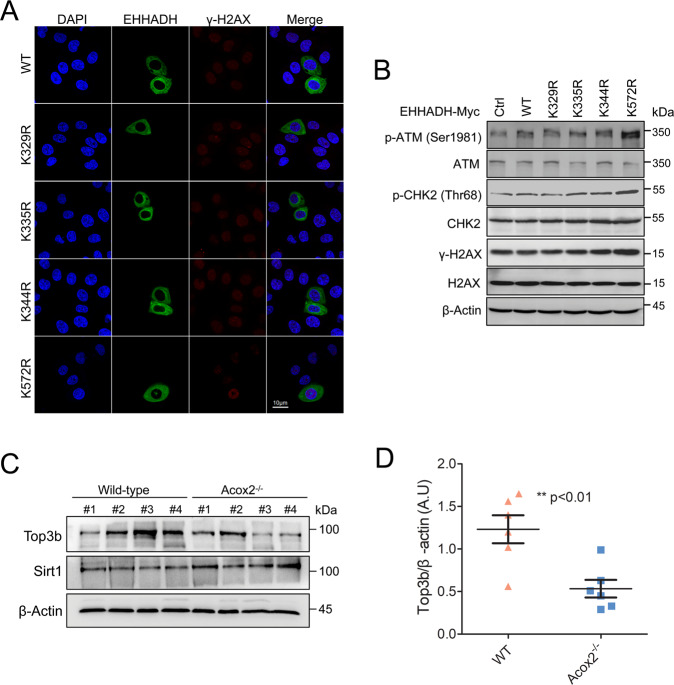
Non-histone Kcr changes caused by Acox2 knockout might be involved in DNA damage responses. **A** Immunofluorescence staining with anti-gammaH2AX antibody in cells expressing Ehhadh WT or (K-R) mutants. **B** Immunoblot analysis of gammaH2AX and pan- or phospho-ATM and CHK2 in cells expressing Ehhadh WT or (K-R) mutants. Beta-actin served as a loading control. **C**, **D** Top3b protein levels were significantly downregulated in the livers of Acox2^−/−^ mice by western Blot (**C**). Quantification data of Top3b expression level when normalized against Beta-actin (**D**). **p value < 0.01 (two-tailed Student’s *t* test; *n* = 6). A.U arbitrary units. Representative image from three independent experiments was displayed.

Previous studies found that loss of TOP3B leads to increased DNA damage and impaired recovery after replication stress [34, 35]. We utilized a anti-gammaH2AX antibody, a sensitive indicator of DNA damage and replication stress, to perform immunofluorescence staining (Fig. 8A) and Western blot (Fig. 8B). We found that gammaH2AX formation is enhanced in cells expressing Ehhadh K572R, when compared with the WT and the other mutants. ATM is the major kinase involved in the phosphorylation of H2AX in response to DNA double-strand breaks (DSBs) [36–38]. Thus, we next evaluate the phosphorylation levels of ATM-CHK2 pathway and found that phosphorylation of ATM (Ser1981) and CHK2 (Thr68) were significantly upregulated in cells expressing Ehhadh K572R when compared with the WT (Fig. 8B). Furthermore, compared with WT mice, Top3b protein levels were significantly downregulated in the liver of Acox2^−/−^ mice by Western Blot (Fig. 8C, D) (*p*-value < 0.01, *n* = 6). These findings suggested that non-histone Kcr changes caused by Acox2 knockout might be involved in DNA damage responses.

## Discussion

Our study highlights the oncogenic property of *Acox2* deficiency in tumor progression as those of our previous [19] and other studies [39]. *Acox2* could be a critical regulator for metabolic homeostasis through post-translational modifications, probably Kcr. Meanwhile, our results demonstrated that Kcr is very prevalent in liver and virtually every enzyme in glycolysis, the tricarboxylic acid (TCA) cycle, and fatty acid metabolism was crotonylated. Importantly, most of downregulated non-histone Kcr sites in *Acox2*^−/−^ mice were located in metabolic enzymes, which extend the scope of metabolism regulation by Kcr to an extent comparable to that of other major posttranslational modifications such as acetylation [40] and ubiquitination [41]. In our study, lymphocytes were diffusely positive for CD20 in Acox2^−/−^ mice. The characteristic of the tumor-infiltrating leukocytes could impact clinical outcome in HCC [42]. Although the role of tumor-infiltrating B cells remains controversial, two previous studies reported that margin-infiltrating CD20(+) B cells correlate with favorable prognosis in HCC [43] and CD20(+) showed a trend towards better DFS [44]. Interaction between tumor-infiltrating B cells and T cells controls the progression of HCC [45]. These findings support the anti-tumor activity of CD20(+) B cells in HCC.

A recent study found that histone crotonylation is crucial for endoderm differentiation by regulating metabolic switch [13]. Differentiation of pluripotent embryonic stem cells to mesoderm and endoderm triggers a metabolic switch from glycolysis to oxidative phosphorylation [46]. This metabolic switch is believed to be important to release stem cells from pluripotency and promote differentiation [47]. Interestingly, this switch is the reverse process for the classic Warburg effect [48]. Thus, Kcr changes caused by Acox2 deficiency might be directly involved in the metabolic switch that is essential for tumor transformation in liver tissues of *Acox2*^−/−^ mice.

Several key peroxisomal enzymes, including Ehhadh, Crot, Scp2, and Hsd17b4, carrying decreased Kcr sites were uniformly upregulated in *Acox2*^−/−^ mice. Studies have found that acetylation can directly affect enzyme stability [49]. For example, the PEPCK1^3KacR^ mutant was more stable than the wild type, whereas the PEPCK1^3KacQ^ mutant remained unstable [49]. Acetylation and crotonylation have similar mechanisms and are both involved in metabolic regulation. Since histone acetyltransferases p300, pCAF, and MOF also have histone crotonyltransferase activity [15, 16], whereas the classic histone deacetylases HDAC3 and SIRT1/2/3 can remove Kcr under different conditions [15, 17], we postulated that changes at non-histone Kcr levels were involved in stability regulation of metabolic enzymes.

A recent study found that CDYL-regulated histone Kcr, mainly H2b (K^12^cr), controls the expression of sex chromosome-linked escaped genes in postmeiotic spermatogenic cells [12]. In the present study, we identified an upregulated Kcr site at H2b (K^86^cr) whereas H2b (K^12^cr) was not significantly changed in *Acox2*^*−/−*^ mice. Thus, investigating the site-specific biological function of H2b (K^86^cr) on liver homeostasis by generating point knock-in mutation would be a potential avenue for understanding these results.

In this study, many downregulated Kcr sites in *Acox2*^−/−^ mice were located in proteins involved in mitochondrial beta-oxidation, including Hadha and Eci1/2, as well as the TCA cycle, including Idh2. Thus, Kcr might provide a vehicle for the cross-talk between peroxisome and mitochondria, which is critical for metabolic regulation. A recent study found that CROT promotes fatty acid metabolism and is involved in mitochondrial dysfunction during vascular calcification [50]. Thus, dysregulated Kcr sites in peroxisomal enzymes could be developed as a potential therapeutic target for human disease.

In this study, Top3b expression is downregulated in the cells expressing Ehhadh K572R as well as in the liver tissues of Acox2^−/−^ mice. Loss of TOP3B was recently reported to increase DNA damage and genome instability [34, 35]. DNA damage sensor gamma-H2AX is increased in preneoplastic lesions of HCC [51]. Mutating the putative Ku70 phosphorylation sites in increased gamma-H2AX and spontaneous induction of HCC [52]. Phosphorylation of Chk2, a central effector of DNA damage response, at threonine68 (Thr68) leads to Chk2 activation and Patients with HCC had increased CHK2 mRNA [53]. Interestingly, a recent study by Carloni et al found that CHK2 overexpression enhances chromosomal instability and HCC progression [54]. In addition, a protein called TAZ in pre-tumor NASH-hepatocytes promotes DNA damage and thereby induce hepatocarcinogenesis [55]. These findings suggested that downregulated Top3b might cause genome instability that is benefit for tumorgenesis in the liver tissues of Acox2^−/−^ mice. On the other hand, non-histone Kcr was also downregulated in the kidney tissues of Acox2^−/−^ mice. Interestingly, quantitative proteomic analysis indicated that protein level of MRE11, a double-strand break repair nuclease, was significantly upregulated in the kidney tissues of Acox2^−/−^ mice (data not shown). Besides self-activation by autophosphorylation at S1981, ATM was activated via the MRE11 complex [56]. Interestingly, MRE11 complex could interact with both ATM and RPA1 [57, 58]. A recent study by Yu et al. found that RPA1 Kcr plays a key role in homologous recombination DNA repair [12]. Thus, upregulated MRE11 might contribute to the DNA damages responses in the kidney tissues of Acox2^−/−^ mice. Over all, our findings provide additional evidences to support the role of non-histone Kcr on DNA damage responses.

The main limitation of our study is that we could not precisely determine the mechanism of different changes in Kcr for histone and non-histone proteins in *Acox2*^−/−^ mice. Thus, some unknown mechanism might regulate this biological process. We postulated that the imbalanced distribution of crotonyl-CoA in the nucleus and cytoplasm is the main cause for this phenomenon. Overall, our results suggest that Acox2 is a regulator for protein post-translational modifications, mainly Kcr, that play important roles on metabolic homeostasis and liver cancer progression in mice.

## Materials and methods

### Generation of *Acox2* knockout mice

*Acox2* knockout (KO) mice were generated by Gempharmatech Co., Ltd. (Nanjing, China) based on the CRISPR-Cas9 technique, as described [59]. C57BL/6n female mice (7–8 weeks old) were used as embryo donors. Briefly, fertilized embryos (zygotes) were collected from the oviducts, and Cas9 mRNA (100 ng/μL) plus sgRNA (50 ng/μL) targeting *Acox2* were mixed and injected into the cytoplasm of fertilized eggs with both pronuclei visible in Chatot–Ziomek–Bavister medium. The injected zygotes were then cultured in Quinn’s Advantage cleavage medium (In Vitro Fertilization, Inc.) containing SCR7 (50 μm; TOCRIS Bioscience, Bristol, UK) for ~24 h, and 18–20 two-cell-stage embryos were transferred into the oviduct of a pseudo-pregnant ICR female mouse at 0.5 dpc (days postcoitus). All mice had access to food and water. All experiments were performed in accordance with Health Guide for the Care and Use of Laboratory Animals. Genomic DNA was extracted from tail tips, and PCR-based genotyping was performed. Sequences of sgRNA and primers are provided in Supplementary data. For all studies, littermate WT and *Acox2*^−/−^ mice derived from heterozygous parents were used.

### Immunofluorescence and immunohistochemistry

For paraffin sections, dewaxing, hydration, and antigen retrieval steps were performed by following standard protocol. After blocking (10% normal goat serum [NGS]), slides were incubated with the primary antibodies as following: anti-pan-Kcr (PTM Biolabs 501 and 502, 1:1000 dilution); anti-Ehhadh (Santa Cruze sc-393123, 1:200 dilution); anti-CK7(Abcam ab181598, 1:2000 dilution); anti-Sox9 (Santa Cruze sc-166505, 1:100 dilution) overnight at 4 °C. The slides were then incubated with the secondary antibody (Goat anti-rabbit or -mouse IgG, Alexa Fluor 488 or 594; Thermo Fisher Scientific, 1:1000 dilution) for 2 h at 25 °C, followed by staining with DAPI (Thermo Fisher Scientific)/PBS for 6 min. Confocal imaging was performed using an SP8 system (Leica), and images were processed using the Leica AF software suite. For immunohistochemistry, anti-CD3 (99940, 1:150 dilution), anti-CD20 (70168, 1:500 dilution) and anti-CD68 (97778, 1:200 dilution) antibodies are from CST and anti-CD138 (Abcam ab128936, 1:500 dilution). Sections were incubated with biotinylated secondary antibodies and horseradish peroxidase avidin D (HRP, Vector). After three 5-min washes of PBS, the sections were developed with DAB kit (Vector) as standard procedures.

### Tandem affinity purification

Tandem affinity purification (TAP)-MS analysis was performed as described [60]. Briefly, MHCC97H and HEK-293T cells (ATCC®ACS-4500™) were grown in DMEM High Glucose (4.5 g/L) (Gibco™), supplemented with 10% FBS (Gibco), 100 IU/mL penicillin and 100 μg/mL streptomycin at 37 °C in a 5% CO_2_ atmosphere. Cells were then transfected with pMCB-SBP-Flag-ACOX2 containing a puromycin resistance marker. Cells were lysed on ice in 0.1% NP40 buffer (20 mM Tris-HCl [pH 8.0], 150 mM NaCl) and centrifuged to remove cell debris; the resulting cell lysates were incubated with streptavidin-conjugated beads (GE Healthcare) for 3 h at 4 °C. The precipitates were washed three times with 0.1% NP40 buffer, 2 times with ddH_2_O, and three times with 50 mmol/L NH_4_CO_3_ and subjected to tryptic digestion at 37 °C overnight. The peptides in the supernatant were collected by centrifugation, dried in a speed vacuum (Eppendorff), and dissolved in NH_4_CO_3_ buffer containing 0.1% formic acid and 5% acetonitrile before being subjected to mass spectrometry.

### Kcr peptide enrichment

To enrich Kcr modified peptides, tryptic peptides were dissolved in NETN buffer (100 mM NaCl, 1 mM EDTA, 50 mM Tris-HCl, and 0.5% NP-40 [pH 8.0]) and then incubated with antibody beads (PTM Bio Inc., Hangzhou) as described [12] at a ratio of 15-μL beads/mg protein at 4 °C overnight. The antibody beads were washed four times with NETN buffer and two times with ddH_2_O. The Kcr peptides were then eluted by adding the elution buffer with 0.1% trifluoroacetic acid. For LC-MS/MS analysis, the resulting peptides were desalted with C18 ZipTips (Millipore) according to the manufacturer’s instructions.

### Tandem mass tagging proteomic analysis

Tandem mass tagging-based proteomics analysis was supported by Jingjie PTM Biolabs (Hangzhou, China) as described [61]. The detailed procedures are provided as Supplementary data. For GO analysis, proteins were classified using GO annotation into three categories: biological process, cellular compartment, and molecular function. For each category, a two-tailed Fisher’s exact test was employed to test the enrichment of differentially modified proteins against all identified proteins. GO with a corrected *p* < 0.05 was considered significant and GO term enrichment was performed using DAVID 6.8.

### Co-immunoprecipitation

pcDNA3.1-MCCC2-Myc (NM_022132) and pEnter-ACOX2-Flag-His (NM_003500) expression vectors were obtained from WZ Biosciences Inc. (Jinan, China) followed by Sanger sequencing validation. HEK-293T was transfected with Myc-tagged MCCC2 and Flag-tagged ACOX2 using Lipofectamine 3000 (Thermo Fisher). After 36 h, cells were lysed in Pierce IP Lysis Buffer (87787, Thermo Fisher) supplemented with proteinase inhibitor. After centrifugation, 200 μg lysates were incubated with anti-Myc-conjugated agarose beads (20168, Thermo Fisher) for immunoprecipitation following standard protocol.

### Site-directed mutagenesis and immunoprecipitation

pCMV6-Ehhadh (Myc-DDK-tagged) and -Crot (Myc-DDK-tagged) mouse ORF clones were purchased from OriGene Technologies Inc (Rockville, USA). The QuickChange site-directed mutagenesis kit (Stratagene; Agilent Technologies) was used to substitute the identified lysine Kcr sites with arginine and generate four mutant Ehhadh variants (K^329^R, K^335^R, K^344^R, and K^572^R) and five mutant Crot variants (K^33^R, K^69^R, K^143^R, K^384^R, and K^489^R), which were confirmed by Sanger sequencing and compared to a reference sequence (RefSeq accession number: NM_023733 for Crot and NM_023737 for Ehhadh). Then HepG2 or 293T cells were transfected with Myc-tagged Crot and Ehhadh constructs followed by anti-Myc immunoprecipitaion as above.

### RNA sequencing

Transcriptome libraries were prepared using a TruSeq RNA Sample Preparation kit (Illumina) and then sequenced using the Illumina HiSeq PE platform at 2 × 151-bp read lengths as previously [62]. The expression level of each transcript was calculated according to fragments per kilobase of exon per million mapped reads. Differentially expressed genes (DEGs) between groups were selected based on a log fold-change >2 and a false discovery rate FDR < 0.05. Gene ontology (GO) functional-enrichment and Kyoto Encyclopedia of Genes and Genomes pathway analyses were performed using GOATOLLS and KOBAS. DEGs were considered significantly enriched when the *p* value was less than 0.05 following Bonferroni’s correction.

### cDNA amplification and RT-qPCR

Total RNA was isolated from HEK-293T cells transfected with mouse Myc-tagged Ehhadh WT or mutant constructs using the RNAprep pure Cell Kit (DP430, Tiangen, Beijing, China) followed by first-strand cDNA synthesis. cDNA was then amplified using PCR with *TOP3B* primers followed by Sanger sequencing. RT-qPCR was performed using the FastFire qPCR PreMix (SYBR Green) (FP207, TIANGEN) on an ABI StepOnePlus instrument. The delta-delta-Ct (ddCt) algorithm was utilized to analyze the relative change in gene expression by using the housekeeping gene GAPDH as the internal reference. The data are presented as the mean ± SD of four independent experiments; each sample was assayed in triplicate in each experiment. RT-qPCR primer sequences are available in Supplementary data.

### Immunoblotting

After undergoing electrophoresis and membrane transfer, the proteins were probed with one of the following primary antibodies in either 5% nonfat milk or 5% bovine serum albumin: anti-pan Kcr (PTM, 501, 502), anti-pan Kac (PTM, 101), anti-pan Ksu (PTM, 401), anti-pan ubiquitin (PTM, 1107), anti-ATM (Abways, CY7229), anti-phospho-ATM (Ser1981) (Abways, CY5111), anti-CHK2 (CST, 6334), anti-phospho-CHK2 (Thr68) (CST, 2197), anti-H2AX (CST, 7631), anti-gammaH2AX (CST, 9718), anti-GAPDH (CST, 8844), anti-Ehhadh (Santa Cruz, sc-393123), TOP3B (Santa Cruz, sc-137238), anti-FH (4567, CST), anti-G6PD (12263, CST), anti-HK2 (2867, CST), anti-Flag (CST, 8146), and anti-Myc (CST, 2278).

### Statistics

Data are presented as individual samples and mean ± SEM. Student’s two-tailed unpaired *t*-test or one-way ANOVA was used to determine statistical significance of differences between WT and mutant groups using GraphPad Prism software v. 5.01 (GraphPad Software, Inc., La Jolla, CA, USA). Differences were significant when *p* values were <0.05.

## Supplementary information

Supplementary DATA

Supplementary Table S1

Supplementary Table S2

Supplementary Table S3

Supplementary Figure S1

Supplementary Figure S2

Supplementary Figure S3

Supplementary Figure S4

Supplementary Figure S5

aj-checklist

## Data Availability

The RNA-sequencing data have been deposited in the Gene Expression Omnibus (GEO) repository under accession number GSE194202. Other data that support the fndings of this study are available from the corresponding authors upon reasonable request.
