# Supplementary material for: Acox2 is a regulator of lysine crotonylation that mediates hepatic metabolic homeostasis in mice
Source: Cell Death Dis. 2022 Mar 29;13(3):279. doi: 10.1038/s41419-022-04725-9 (PMC8964741; doi:10.1038/s41419-022-04725-9)
Supplement: Supplementary file 1 — Supplementary DATA [file 41419_2022_4725_MOESM1_ESM.docx]

**SUPPLEMENTARY FIGURE LEGENDS**

***Figure S1. Acox2 loss induces inflammatory infiltration and liver cancer.*** (A) Schematic representation for generation of *Acox2^-/-^* mice by CRISPR/Cas9 system. (B) Hematoxylin and eosin (H&E) staining revealed that inflammatory infiltration (yellow arrow) and liver cancer in the liver tissues of *Acox2^−/−^* mice at the postnatal age of 8–9 months. Heterogeneity was observed in tumor lesions of Acox2^-/-^ mice livers (yellow circus). Representative images were displayed. Scale bar, 100 μm, 20 μm and 10 μm.

***Figure S2. The specificity of anti-Kcr antibody (PTM 501).*** (A) The distribution and levels of Kcr in two cultured cell lines including iPSC (induced pluripotent stem cells) and HepG2. (B-C) Compared to cells without treatment (Ctrl), Kcr levels were deceased in cells treated with P300 inhibitor (C646) whereas Kcr signals were upregulated in cells treated with HDACI (FK228) by immunoblotting (B) and immunofluorescence assay (C). HepG2 cells were then treated with p300 inhibitor (C646 and HAI) and HDACI (FK228, TSA and RGFP966) with indicated concentration, respectively.Representative images were displayed. Scale bar, 8 μm.

***Figure S3. The distribution of Kcr in tumor lesions of Acox2^-/-^ mice livers.*** Immunohistochemical staining showed Kcr signals were mostly concentrated in the nucleus of tumor cells when compared with adjacent normal cells in *Acox2^−/−^* mice. Boxed areas are enlarged to show the merged region between anti-Kcr and DAPI staining. Scale bar, 5 μm.

***Figure S4. Kcr levels were increased in areas of ductular reaction.*** (A) Kcr signal intensity in cytoplasm of normal liver cells in Acox2^-/-^ mice was significantly decreased when compared with WT mice. (B) H&E and Sirius red staining showing the proliferation of reactive bile ducts. (C) Elevated Kcr signals in areas of ductular reaction in Acox2^-/-^ mice. (D) Co-localizations of Kcr signal and ductal marker Sox9 were observed by double immunostaining. Representative images were displayed.

***Figure S5. Transcriptome analysis on 293T cells expressing Ehhadh WT and K-R mutants.*** (A) PCA analysis based on 3 biological replicates effectively separated K572R from Ehhadh WT or the other mutants. (B) Hierarchical clustering results and heatmap representation of (differentially expressed genes) DEGs profiles are shown for each group.

**SUPPLEMENTARY TABLES**

Table S1. Summary of top 30 candidate ACOX2-interacting proteins in MHCC97H cell line.

Table S2. Summary of top 30 candidate ACOX2-interacting proteins in HEK-239T cell line.

Table S3. Top50 downregulated Kcr sites on non-histone proteins in *Acox2^-/-^* mice.

**SUPPLEMENTARY METHODS**

**Tandem Mass Tagging Proteomics Analysis**

Tandem mass tagging (TMT) proteomic analysis was supported by Jingjie PTM Biolabs (Hangzhou, China) as described (1, 2). Briefly, the liver tissue sample was ground in liquid nitrogen into powder and then transferred to a 5-mL centrifuge tube. After that, four volumes of lysis buffer (8 M urea, 1% Protease Inhibitor Cocktail) were added to the powder, followed by sonication three times on ice using a high intensity ultrasonic processor (Scientz). For digestion, the protein solution was reduced with 5 mM dithiothreitol for 30 min at 56 °C and alkylated with 11 mM iodoacetamide for 15 min at room temperature in dark. The protein sample was then diluted by adding 100 mM TEAB to urea concentration less than 2 M. Finally, trypsin was added at 1:50 trypsin-to-protein mass ratio for the first digestion overnight and 1:100 trypsin-to-protein mass ratio for the second 4 h-digestion. After trypsin digestion, the peptides were desalted, using the Strata X C18 SPE column (Phenomenex), and vacuum-dried. The peptides were reconstituted in 0.5 M TEAB and processed according to the manufacturer’s protocol (TMT kit/iTRAQ kit). Briefly, one unit of TMT/iTRAQ reagent was thawed and reconstituted in acetonitrile. The peptide mixtures were then incubated for 2 h at room temperature and pooled, desalted, and dried by vacuum centrifugation. The tryptic peptides were fractionated by high pH reverse-phase HPLC using Thermo Betasil C18 column (5-μm particles, 10 mm ID, 250 mm length). Briefly, peptides were first separated under a gradient of 8%–32% acetonitrile (pH 9.0) over 60 min into 60 fractions. Then, the peptides were combined into 6 fractions and dried by vacuum centrifuging.

For LC-MS/MS analysis, the tryptic peptides were dissolved in 0.1% formic acid (solvent A), directly loaded onto a home-made reversed-phase analytical column (15-cm length, 75 μm i.d.). The gradient comprised an increase from 6% to 23% of solvent B (0.1% formic acid in 98% acetonitrile) over 26 min, 23%–35% in 8 min, and climbing to 80% in 3 min then holding at 80% for the last 3 min, all at a constant flow rate of 400 nL/min on an EASY-nLC 1000 UPLC system. The peptides were subjected to NSI source followed by tandem mass spectrometry (MS/MS) in Q ExactiveTM Plus (Thermo) coupled online with the UPLC. The electrospray voltage applied was 2.0 kV. The m/z scan range was 350 to 1800 for the full scan, and intact peptides were detected in the Orbitrap at a resolution of 70,000. Peptides were then selected for MS/MS using NCE setting as 28 and the fragments were detected in the Orbitrap at a resolution of 17,500. A data-dependent procedure was followed that alternated between one MS scan followed by 20 MS/MS scans with 15.0s dynamic exclusion. Automatic gain control (AGC) was set at 5E4. Fixed first mass was set as 100 m/z.

The resulting MS/MS data were processed using Maxquant search engine (v.1.5.2.8). Tandem mass spectra were searched against the Uniprot database with the reverse decoy database. Trypsin/P was specified as cleavage enzyme, allowing up to 4 missing cleavages. The mass tolerance for precursor ions was set as 20 ppm in the First search and 5 ppm in the Main search. Mass tolerance for fragment ions was set as 0.02 Da. Carbamidomethyl on Cys was specified as fixed modification and acetylation modification and oxidation on Met were specified as variable modifications. FDR was adjusted to <1% and minimum score for modified peptides was >40.

For Gene Ontology (GO) analysis, proteins were classified by GO annotation into three categories: biological process, cellular compartment, and molecular function. For each category, a two-tailed Fisher’s exact test was used to test the enrichment of differentially modified proteins against all identified proteins. A GO with a corrected *p* < 0.05 was considered significant. The Kyoto Encyclopedia of Genes and Genomes (KEGG) database was used to identify enriched pathways using a two-tailed Fisher’s exact test to test the enrichment of differentially modified proteins against all identified proteins. The pathway with a corrected *p* < 0.05 was considered significant. These pathways were classified into hierarchical categories according to the KEGG website. For Protein Domain analysis, domain functional description of identified proteins was annotated by InterProScan based on protein sequence alignment method, and the InterPro domain database was used. For protein–protein interaction (PPI) network analysis, the STRING database was used and protein network data were visualized in R package “networkD3”. GO term and KEGG pathway enrichment were performed using DAVID 6.8.

SgRNA for Acox2 knockout generation

| sgRNA | SgRNA sequences (5’-3’) | PAM |
| --- | --- | --- |
| S1 | CCACTCCTGCAGAGCGTGAC | AGG |
| S2 | CTCGCCTCATGGTGTTGGAA | AGG |
| S3 | CATGTTAGACCCTGGTGCGC | TGG |
| S4 | GTCTAGGCCCCAAGTTTGCA | AGG |

Primers for *Acox2^-/-^* mice genotyping

| Primer name | Primer sequences | Product size |
| --- | --- | --- |
| Acox2-KO-tF1 | GGAGAAGAGTTGGTAAGCAGAGTG | KO:844bp  Wt: 16014bp |
| Acox2-KO-tR1 | TGTTGAAGAGTTCAAGTGAGGGAC |  |
| Acox2-Wt-tF1 | CATGACCCGAGATGAGCTATATG | KO: none  Wt:457bp |
| Acox2-Wt-tF1 | TACCATGTCCCAGCTCTGTCTG |  |

Primers for real-time qPCR

| Primer name | Primer sequences |
| --- | --- |
| TOP3B-F | 5’-GCCCCGAAACTGGAGTGT-3’ |
| TOP3B-R | 5’-CCGTTCAGCCCTTTGTGT-3’ |
| GAPDH-F | 5’-GGAAGGTGAAGGTCGGAGT-3’ |
| GAPDH-R | 5’-ATCGCCCCACTTGATTTTG -3’ |
