# Supplementary material for: Acox2 is a regulator of lysine crotonylation that mediates hepatic metabolic homeostasis in mice
Source: Cell Death Dis. 2022 Mar 29;13(3):279. doi: 10.1038/s41419-022-04725-9 (PMC8964741; doi:10.1038/s41419-022-04725-9)
Supplement: Supplementary file 2 — Supplementary Table S1 [file 41419_2022_4725_MOESM2_ESM.pdf]

Table S1. Summary of top 30 candidate ACOX2-interacting proteins in MHCC97H cell line.

Description	Score	Coverage	Unique Peptides	Peptides	PSMs	AAs	MW [kDa]	calc. pI
PLEC	11788.38	69.41	298	361	512	4684	531.466	5.96044922
ACACA	10151.29	79.92	167	192	458	2346	265.3849	6.36669922
PYC	7873.047	70.71	85	85	323	1178	129.5514	6.84423828
MCCC1	3966.671	66.34	43	43	144	725	80.42193	7.78173828
MCCC2	3097.163	82.24	47	47	125	563	61.29441	7.67919922
ACACB	2740.218	40.36	30	84	135	2458	276.365	6.49365234
Q5HY54	2178.23	39.05	1	71	89	2607	276.3778	6.04931641
K2C8	2172.242	60.46	37	44	95	483	53.67114	5.59228516
Q60FE5	2146.237	38.78	1	71	89	2620	278.0528	6.06201172
LMNA	2054.172	68.22	13	59	86	664	74.09471	7.02001953
PCCA	1888.514	59.89	40	40	70	728	80.0081	7.51806641
ACTB	1883.752	75.2	1	29	72	375	41.70973	5.47802734
ACTG	1816.061	75.2	1	29	69	375	41.76579	5.47802734
TOP2A	1771.077	36.77	50	64	81	1531	174.2758	8.71923828
K1C18	1750.986	62.09	27	29	49	430	48.02854	5.45263672
K2C1	1553.369	50.31	32	38	55	644	65.999	8.11865234
PRKDC	1528.983	18.24	64	64	65	4128	468.7879	7.12255859
Q5TCI8	1486.316	70.26	1	47	71	491	55.72859	7.02001953
CLH1	1483.447	36.42	49	49	61	1675	191.4925	5.69384766
DHX9	1427.82	36.06	40	40	55	1270	140.8691	6.84423828
ROA2	1425.418	72.52	27	31	54	353	37.40673	8.95361328
H4	1412.957	62.14	17	17	63	103	11.36038	11.355957
H0YGH5	1344.366	56	1	45	72	859	97.41944	6.44287109
TOP2B	1334.145	32.16	39	53	65	1626	183.1525	8.00146484
K1C19	1246.323	76	24	35	52	400	44.07912	5.13525391
PCCB	1239.868	59.37	28	28	48	539	58.17875	7.63525391
CH60	1205.854	52.36	28	28	39	573	61.01639	5.87158203
BAZ1B	1203.69	34.93	45	46	59	1483	170.7957	8.48486328
B9A067	1173.344	52.04	41	41	58	711	78.92499	7.06396484
HNRPM	1132.167	55.34	24	40	57	730	77.46432	8.70458984
