# Supplementary material for: Acox2 is a regulator of lysine crotonylation that mediates hepatic metabolic homeostasis in mice
Source: Cell Death Dis. 2022 Mar 29;13(3):279. doi: 10.1038/s41419-022-04725-9 (PMC8964741; doi:10.1038/s41419-022-04725-9)
Supplement: Supplementary file 3 — Supplementary Table S2 [file 41419_2022_4725_MOESM3_ESM.pdf]

Table S2. Summary of top 30 candidate ACOX2-interacting proteins in HEK-239T cell line.

Description	Score	Coverage	Unique Peptides	Peptides	PSMs	AAs	MW [kDa]	calc. pI
ACACA	10687.99	75.11	161	184	460	2346	265.3848622	6.366699
PYC	4296.195	66.04	48	71	168	1178	129.5514175	6.844238
Q5HY54	3866.669	55.62	2	112	151	2607	276.3777877	6.049316
Q60FE5	3781.642	54.5	1	111	150	2620	278.0527705	6.062012
ACOX2	3312.037	70.34	47	47	127	681	76.77798604	7.562012
PCCA	3167.01	62.23	50	50	110	728	80.00810157	7.518066
FAS	2668.819	42.17	1	84	105	2511	273.2543005	6.442871
A0A0U1RQF0	2660.844	42.21	1	84	105	2509	273.0261895	6.468262
MCCB	2649.145	78.15	40	40	98	563	61.29441059	7.679199
ACACB	2644.95	34.87	54	69	121	2458	276.3650244	6.493652
HS71B	2628.896	64.59	25	45	98	641	70.00904046	5.655762
MYH9	2587.132	46.12	70	87	104	1960	226.3916023	5.60498
PCCB	2328.574	72.54	36	36	73	539	58.17875253	7.635254
MCCA	2283.7	58.21	35	35	92	725	80.42193397	7.781738
PRKDC	2218.262	23.21	87	87	97	4128	468.7879323	7.122559
TPR	2157.351	39.61	81	82	96	2363	267.1311248	5.020996
HS90A	1860.569	54.92	28	43	69	732	84.60668521	5.020996
TBB5	1857.874	73.42	4	27	74	444	49.63897361	4.894043
HS90B	1827.807	58.29	27	44	67	724	83.21210592	5.033691
E9PRE7	1818.107	61.96	1	24	72	489	53.37762333	7.664551
HSP7C	1779.652	58.67	4	42	72	646	70.8542269	5.516113
RBP2	1764.063	25.16	63	63	73	3224	357.9740692	6.20166
PARP1	1688.204	56.11	62	62	76	1014	113.0123999	8.880371
TCPB	1664.902	76.64	41	41	57	535	57.45213278	6.455566
TCPQ	1631.91	76.64	44	44	59	548	59.58250903	5.60498
CH60	1629.053	72.95	37	38	48	573	61.01638505	5.871582
ACTB	1552.77	73.6	1	28	59	375	41.70973209	5.478027
K1C10	1537.885	51.2	25	29	36	584	58.79169631	5.211426
TBB2B	1509.037	74.16	2	27	67	445	49.92094649	4.894043
TBA1B	1499.553	58.98	4	27	57	451	50.11960685	5.059082
