# Supplementary material for: Acox2 is a regulator of lysine crotonylation that mediates hepatic metabolic homeostasis in mice
Source: Cell Death Dis. 2022 Mar 29;13(3):279. doi: 10.1038/s41419-022-04725-9 (PMC8964741; doi:10.1038/s41419-022-04725-9)
Supplement: Supplementary file 4 — Supplementary Table S3 [file 41419_2022_4725_MOESM4_ESM.pdf]

Table S3. Top50 downregulated Kcr sites of non-histone proteins in *Acox2*$^{-/-}$ mice

Gene name	Amino acid	Kcr sites	Kcr Ratio (Acox2$^{-/-}$_vs_WT)	*p*-value
Ehhadh	K	329	0.284	0.00041869
Crot	K	33	0.391	0.0060388
Crot	K	69	0.426	0.0117047
Gsta1	K	141	0.428	0.025678
Ehhadh	K	344	0.448	0.00170182
Crot	K	489	0.494	0.00040407
Crot	K	384	0.522	0.000118643
Scp2	K	524	0.523	0.029743
Dhrs4	K	204	0.528	0.0025608
Crot	K	226	0.544	0.00171897
Hadha	K	190	0.561	0.028375
Prkar2a	K	247	0.574	0.046596
Gsta1	K	138	0.575	0.0085772
Hacl1	K	354	0.607	0.00140474
Scp2	K	211	0.619	0.0133188
Cul1	K	676	0.628	0.028416
Cyp2a12	K	385	0.628	0.04368
Ces1e	K	305	0.632	0.031515
Egfr	K	881	0.634	0.047516
Abcd3	K	529	0.636	0.0169951
Hsd17b4	K	57	0.639	0.0035998
Eci1	K	229	0.639	0.0150012
Gm4952	K	169	0.64	0.022925
Acaa1a	K	292	0.642	0.000178874
Retsat	K	374	0.648	0.0051225
Etfa	K	139	0.649	0.039101
Hmgcs2	K	327	0.649	0.024555
Gm4952	K	183	0.65	0.020535
Etfdh	K	152	0.656	0.0118168
Hsd17b4	K	81	0.657	0.00110493
Cyp2c70	K	321	0.659	0.03338
Anxa6	K	240	0.662	0.0176004
Uox	K	118	0.664	0.0090552
Crot	K	143	0.664	0.0020635
Uqcrq	K	33	0.672	0.0186755
Cyp2c50	K	383	0.674	0.048644
Ces1d	K	480	0.684	0.0079988
Hsd17b4	K	68	0.684	0.0149612
Baat	K	40	0.686	0.041424
Eci2	K	90	0.695	0.0038555
Otc	K	80	0.697	0.030537
Idh2	K	67	0.699	0.037261
Adh1	K	186	0.699	0.049279
Cpt1a	K	180	0.701	0.0157646
Pxmp2	K	19	0.708	0.037519
Slc25a22	K	188	0.708	0.0162396
Cat	K	77	0.709	0.00084078
Lactb2	K	209	0.709	0.030643
Lonp2	K	212	0.712	0.0159633
Fabp1	K	20	0.714	0.035943
