# Supplementary figures and images for: Acox2 is a regulator of lysine crotonylation that mediates hepatic metabolic homeostasis in mice
Source: Cell Death Dis. 2022 Mar 29;13(3):279. doi: 10.1038/s41419-022-04725-9 (PMC8964741; doi:10.1038/s41419-022-04725-9)

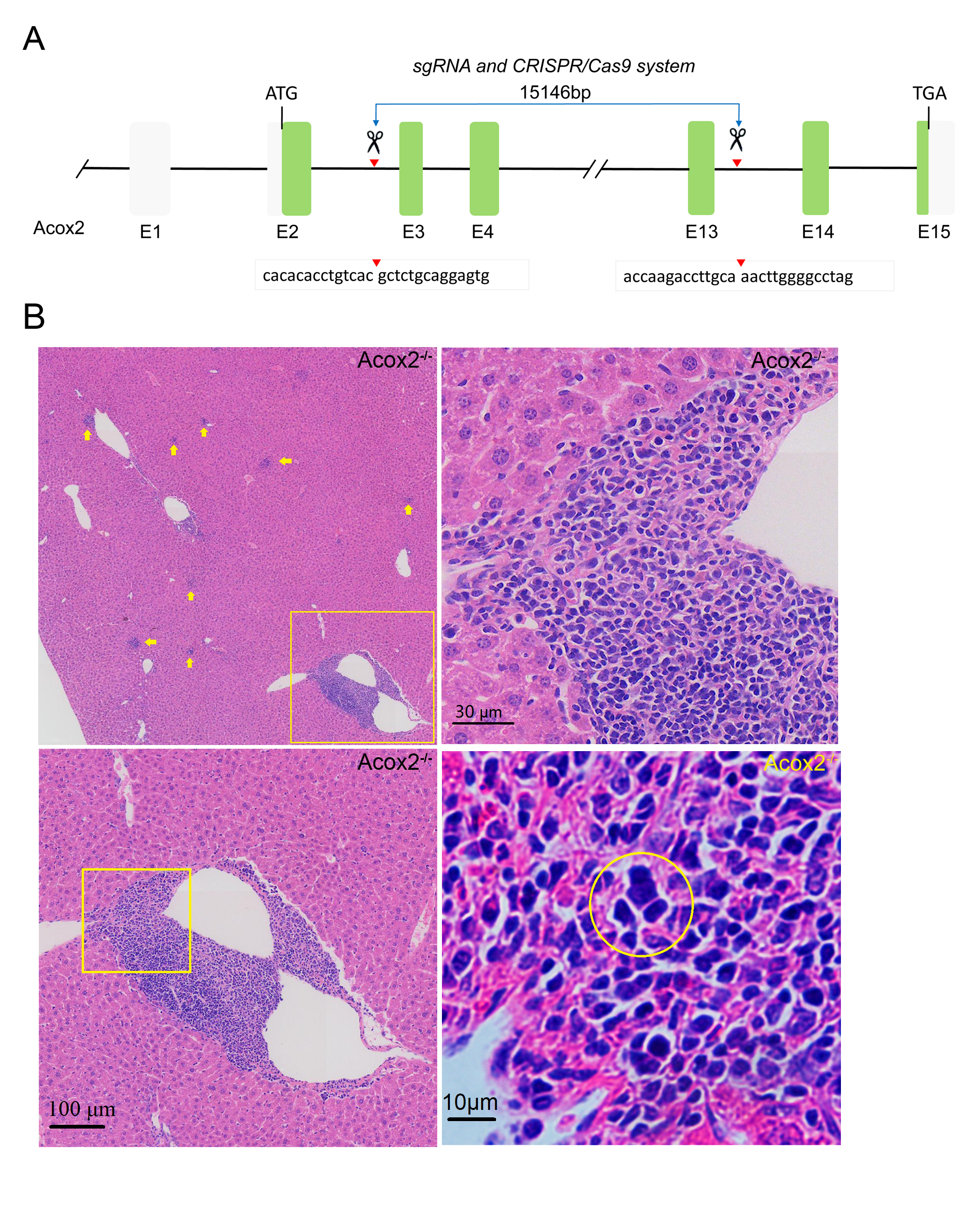

Supplement: Supplementary file 5 — Supplementary Figure S1 [file 41419_2022_4725_MOESM5_ESM.tif]

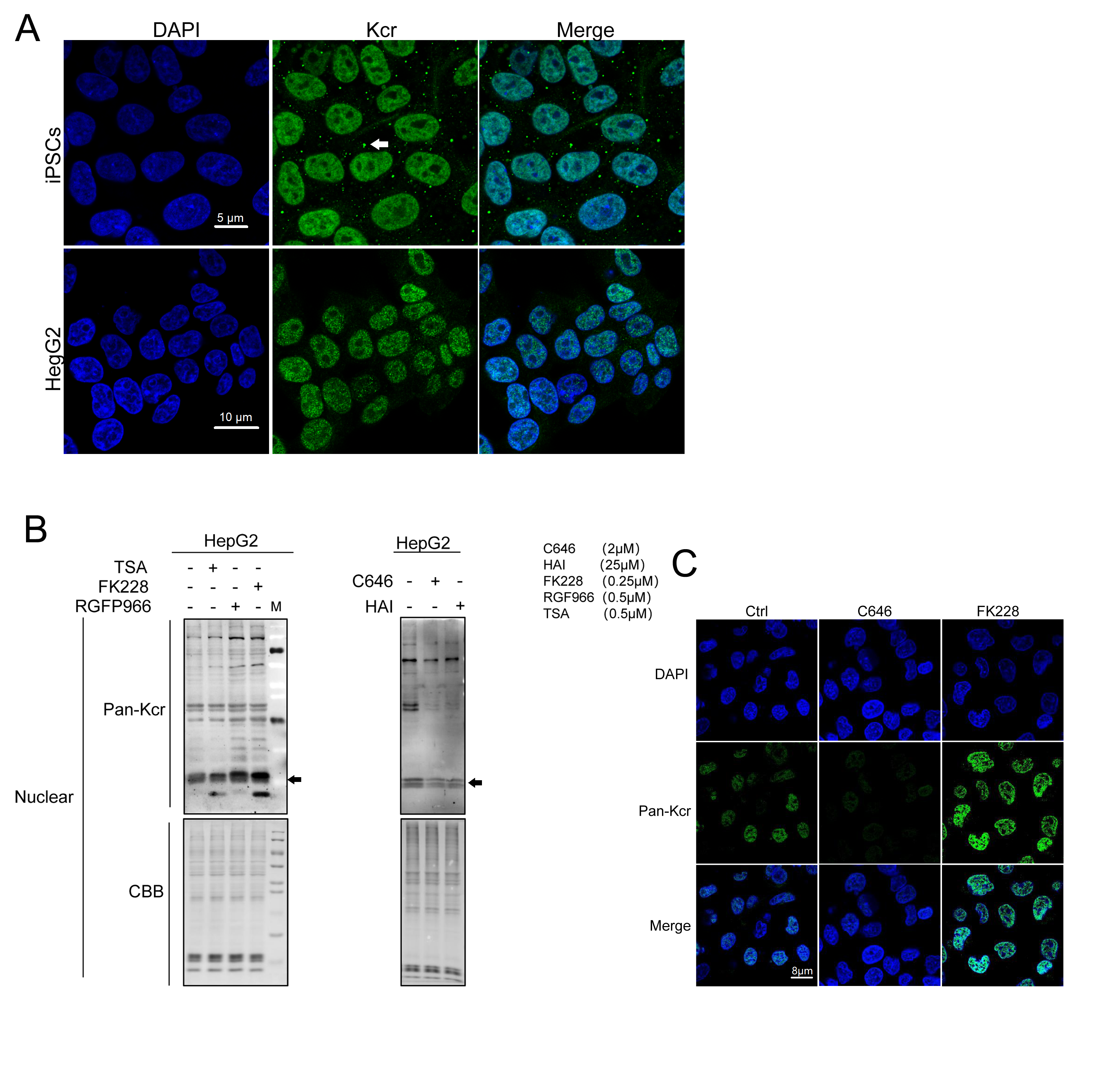

Supplement: Supplementary file 6 — Supplementary Figure S2 [file 41419_2022_4725_MOESM6_ESM.tif]

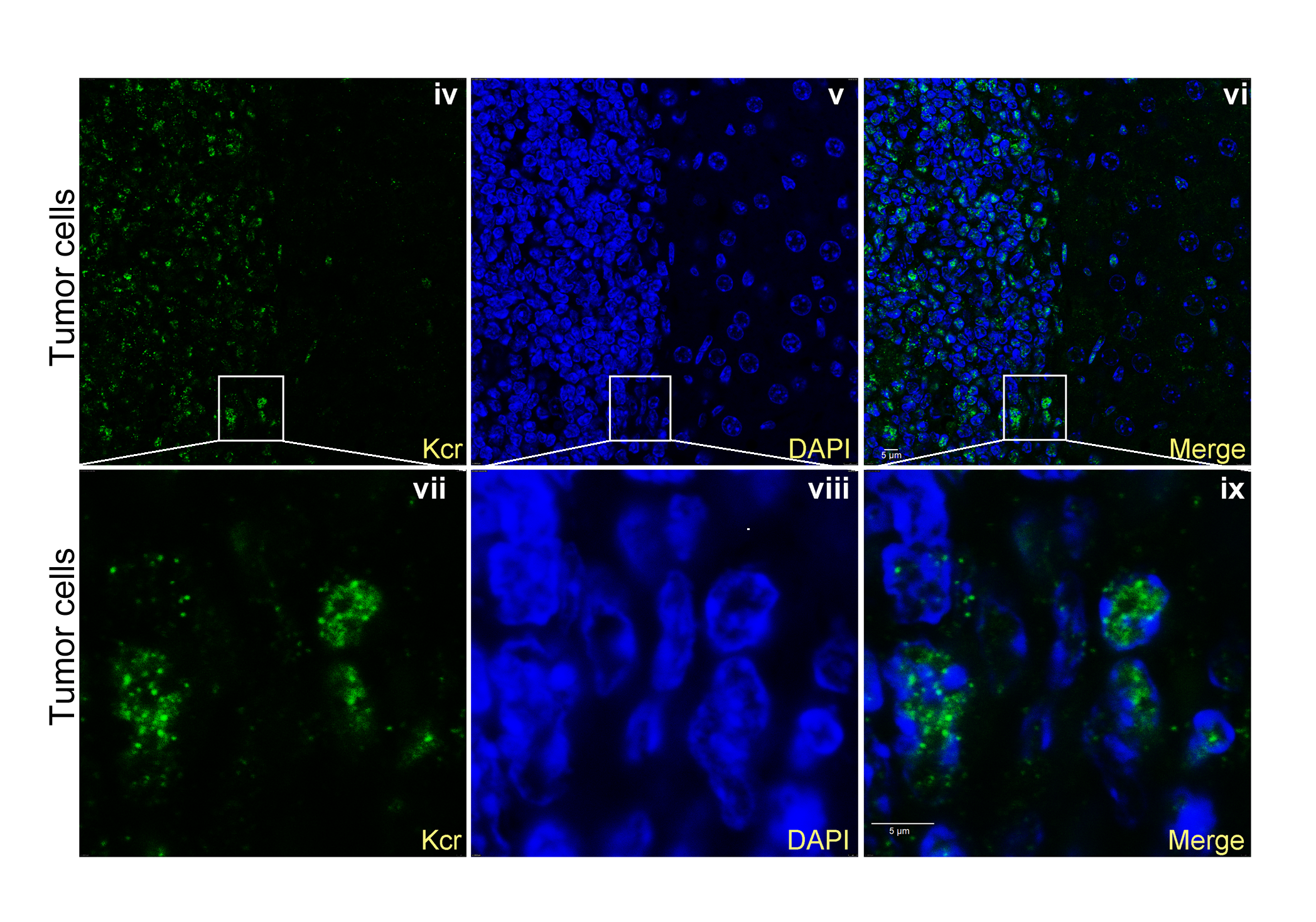

Supplement: Supplementary file 7 — Supplementary Figure S3 [file 41419_2022_4725_MOESM7_ESM.tif]

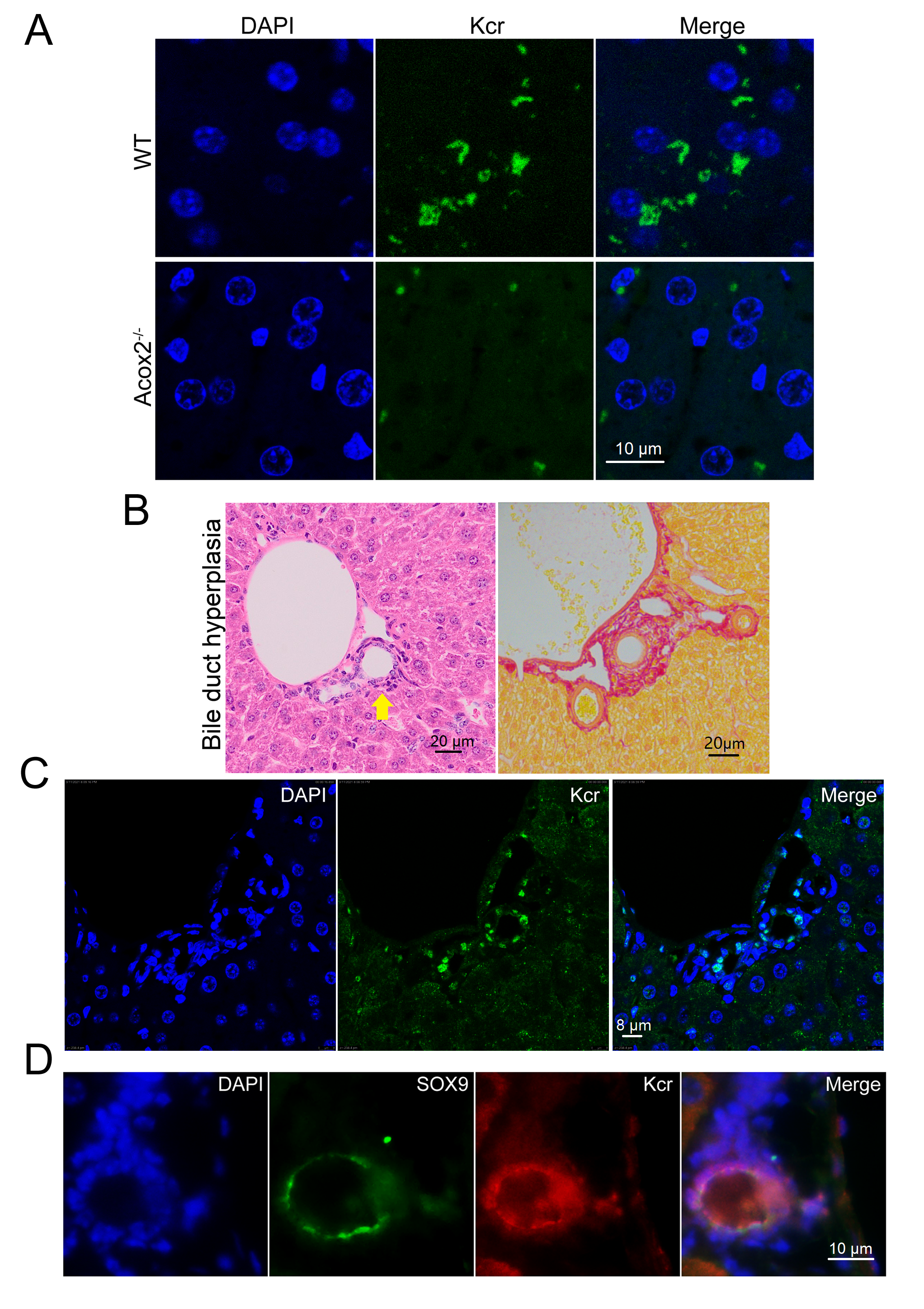

Supplement: Supplementary file 8 — Supplementary Figure S4 [file 41419_2022_4725_MOESM8_ESM.tif]

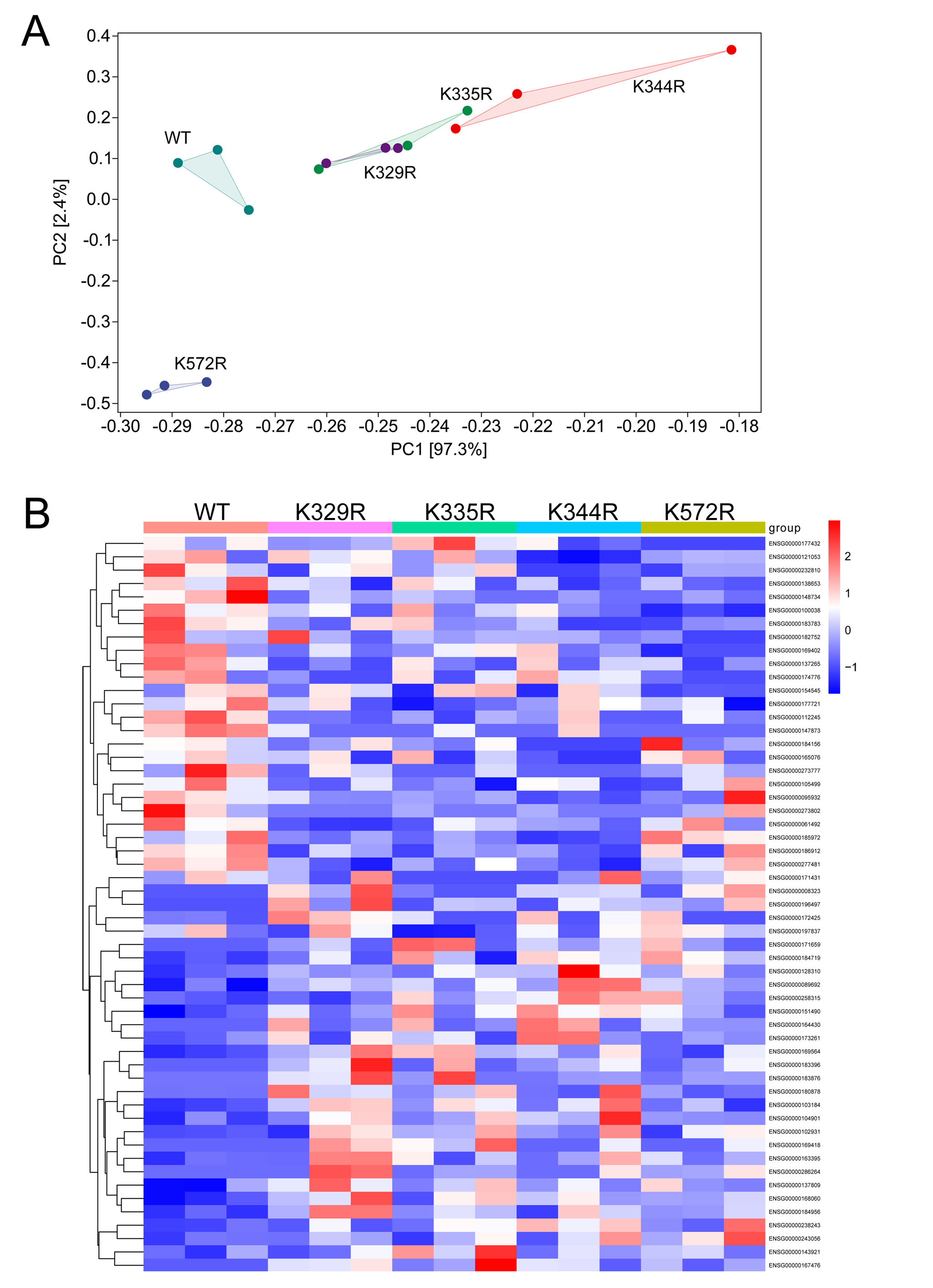

Supplement: Supplementary file 9 — Supplementary Figure S5 [file 41419_2022_4725_MOESM9_ESM.tif]
